# Behavioral Profiles and Attitude toward Condom Use among College Students in Southwest Ethiopia

**DOI:** 10.1155/2020/9582139

**Published:** 2020-09-23

**Authors:** Tewodros Yosef, Tadesse Nigussie

**Affiliations:** Department of Public Health, College of Medicine and Health Sciences, Mizan-Tepi University, Mizan Teferi, Ethiopia

## Abstract

**Background:**

Unsafe sexual behavior among adolescents still represents a public health challenge. To have safe sex, effective condom utilization is needed. Condom use remains relatively low among adolescents in sub-Saharan Africa. Even though adolescents have good knowledge about condom use, they are still engaged in risky sexual behavior.

**Objective:**

To assess condom use and attitude toward condom use among college students in southwest Ethiopia.

**Methods:**

A cross-sectional study was conducted among 453 students at Mizan-Aman Polytechnic College in southwest Ethiopia. Data were collected using a structured self-administered questionnaire. The collected data were entered using EpiData version 4.2.0.0 and analyzed using SPSS version 20 statistical software. Binary logistic regression was computed. Independent variables with a *p* value of less than 0.05 in the multivariable logistic regression model were considered significant.

**Results:**

Of the 453, 180 were sexually active. Among those sexually active, 119 (66.1%) used condoms in their last sexual intercourse. The proportion of positive attitude toward condom use was 53.4%, and the mean attitude score for condom use was 28.6 (±9.99 SD) ranging from 10 to 50. The study also found that being male (AOR = 1.77, 95% CI [1.19-2.65]), rural resident (AOR = 2.20, 95% CI [1.47-3.30]), ever had sex (AOR = 1.87, 95% CI [1.23-2.85]), and knowledge of STIs (AOR = 1.66, 95% CI [1.10-2.51]) were factors associated with a positive attitude toward condom use.

**Conclusion:**

The proportion of positive attitude toward condom use among college students in Ethiopia was low. The study also found that being male, rural resident, ever had sex, and knowledge of STIs were factors associated with a positive attitude toward condom use. Therefore, strengthening information, education, and communication (IEC) on condom self-efficacy; providing condoms on campuses; and imparting education about sexually transmitted infections for young adults are central for improving condom use and attitudes toward condom use. Besides, inculcating sexual and reproductive health in the educational curriculum plays paramount importance.

## 1. Introduction

Sexually transmitted diseases (STDs) are current public health concerns [1] and are considered a major global cause of acute illness, infertility, long-term disability, and death, with serious medical and psychological consequences for millions of men and women. Its prevalence is high in developing countries, resulting in substantial losses of productivity among individuals and communities, where the majority of the population is less than 40 years of age [2, 3]. STDs are associated with increased transmission of human immunodeficiency virus/acquired immune deficiency syndrome (HIV/AIDS) and poor reproductive and sexual health [4].

Globally, a condom is considered an important method for the prevention of sexually transmitted infections, especially HIV/AIDS, and a means to prevent unwanted pregnancy [5–8]. Condom use among young adults has been increasing over the past two decades. However, its magnitude varies from more than 80% in some Latin American and European countries to less than 30% in some African countries [9]. STIs are mostly transmitted through sexual intercourse through unsafe sex [10]. Unsafe sexual behavior among young adults still represents a public health challenge [11–13]. To have safe sex, effective condom utilization is needed [14]. For condoms to be effective, they have to be consistently and continuously used. Despite the various promotion methods, condom use remains relatively low among adolescents in sub-Saharan Africa [15–17].

Different studies conducted globally regarding the prevalence of condom use reported that 53% and 50.3% in Tanzania [12, 18], 24% in the Democratic Republic of Congo [19], 38.6% in Nigeria [6], 68.3% in Ethiopia [20], 16.6% in Kenya [21], 12% in Iraq [5], and 51% in Spain [22] of the participants used condoms during their last sexual intercourse.

Having good knowledge about condoms does not guarantee its utilization. Young adults with good knowledge about condoms are still engaged in risky sexual behavior (unsafe sex) [15]. Condom use for safe sex is mainly influenced by attitudes toward condom use [22]. Studies conducted elsewhere regarding attitude toward condom use reported that 52% in the Democratic Republic of Congo [19] and 68.8% in Iraq [5] had positive attitudes toward condom use. The factors associated with attitude toward condom use are diverse and may include age, sex, residence, marital status, religion, educational status, and knowledge of STIs [15, 16, 18, 23–26].

A dramatic increase in human immunodeficiency virus (HIV) infection among young people has become alarming in Ethiopia [27]. Even though premarital sex relationships are widely discouraged in Ethiopia, young people in colleges become highly engaged in risky sexual behaviors [28, 29] due to a sense of independence from restrictions and parental impact. Studies were done previously regarding the magnitude of condom use [20] and the descriptive nature of attitude toward condom use [30] in Ethiopia. But no study has clearly shown the attitude toward condom use and its associated factors in these populations. Therefore, this study is aimed at assessing condom use and attitude toward condom use among college students in southwest Ethiopia.

## 2. Materials and Methods

### 2.1. Study Design, Setting, and Period

A cross-sectional study was conducted at Mizan-Aman Polytechnic College (MAPtC) students from April 01 to 30, 2018. MAPtC is found at 585 km southwest of Addis Ababa, the capital city of Ethiopia. The college was established in 2005. The college teaches students in ten departments, with five/four levels for each department. The departments were garment and textile, automotive, road construction, water and sanitation, information communication technology, building electrical installation, electrotechnology, masonry construction, general metal fabrication, and surveying technology. The college had a total of 1810 students (920 male and 890 female) during the study period. All students were not in dormitories (live outside of the college) [31].

### 2.2. Populations

The source population includes all regular college students, who attended their class during the study period. The study population includes randomly selected students who studied during the study period.

### 2.3. Sample Size Determination and Sampling Method

The sample size was determined using a single population proportion formula using the following assumptions: the expected proportion of positive attitude toward condom use was 83.6% [30], 5% precision level, 95% confidence interval, 10% for nonresponse compensation, and a design effect of 2. The final computed sample size was 464. A stratified random sampling technique was used to select 464 regular students. In Mizan-Aman Polytechnic College, there were ten departments with five/four levels for each department. The departments were stratified based on levels (levels I-V). For each level, the sample size is proportionally allocated. The potential participants were selected using systematic random sampling.

### 2.4. Data Collection Instrument and Procedures

The data were collected through structured self-administered and pretested questionnaire. The questionnaire is composed of sociodemographic characteristics, attitude questions regarding condom use, and behavioral profiles. The questionnaire was developed by reviewing relevant literatures in English, then translated into the local language (Amharic), and back-translated into English to check the consistency by an independent translator. The training was given to data collectors and supervisors concerning the objective and process of data collection to discuss the presence of an ambiguous question in the questionnaire.

### 2.5. Study Variables

The dependent variable was attitude toward condom use. The independent variables were age, sex, residence, marital status, academic level, ever had sex, number of sexual partners, and knowledge about STIs.

### 2.6. Operational Definitions

*Ever had sex (sexually active)* was defined as a study subject who had at least one sexual intercourse before the study. *Multiple sexual partners* was defined as having more than one sexual partner. *Practice condom use* was defined as using a condom by respondents in their last sexual intercourse. *Positive attitude toward condom use* was defined as when respondents scored the mean and above the value of attitude toward condom-related questions, otherwise negative attitude toward condom use.

### 2.7. Data Processing and Analysis

The completeness and consistency of the data were checked, coded, and entered into EpiData 4.2.0.0. The data were exported to SPSS version 20 statistical software for further analysis. Descriptive and summary statistics were carried out. The results are presented in tables and numerical summary measures such as mean and standard deviation (SD). Bivariate and multivariable logistic regression analyses were used to identify variables associated with attitude toward condom use. Independent variables with a *p* value of less than 0.25 in bivariate logistic regression were included in the multivariable logistic regression model. Finally, variables with a *p* value < 0.05 in the multivariable logistic regression model were considered significantly associated with the dependent variable. The Hosmer-Lemeshow goodness-of-fit test indicated (*p* = 0.740) that the model was good enough to fit the data well.

## 3. Results

### 3.1. Sociodemographic Characteristics

Of the 464, 453 students filled the questionnaire with a response rate of 97.6%. The mean age of respondents was 19.95 (±2 SD) ranging from 18 to 30 years. The majority of the respondents were male (53.6%), single (88.1%), and orthodox Christian followers (54.7%). More than half of the study participants were from urban residence (57.6%), and 52.1% were in the age group of 20 years and above (Table 1).

### 3.2. Behavioral Profiles

Almost one-tenth (10.4%) of the respondents were cigarette smokers. One hundred seven (23.6%) and 172 (38%) of the respondents were alcohol drinkers and watching pornography at least once in their lifetime, respectively. Of the 180 sexually active, 119 (66.1%) and 82 (45.6%) used condoms in their last sexual intercourse and had multiple sexual partners, respectively (Table 2).

### 3.3. Attitude Score of Condom Use

The mean attitude score of respondents was 28.6 (±9.99 SD) with a range of 10 to 50. Two hundred forty-two (53.4%) respondents had a positive attitude toward condom use. Two hundred twenty-three (49.2%) of the participants agreed with “condoms as an effective method of preventing pregnancy.” One hundred ninety-one (42.2%) of the study subjects agreed that “condom reduces sexual pleasure.” The majority of 212 (46.8%) study subjects agreed with “feeling protected while using a condom.” One hundred fifty-nine (35.1%) of the study subjects agreed that “condoms are too expensive to buy.” Ninety-six (21.2%) and 133 (29.9%) respondents disagreed with “condoms are suitable for casual sex” and “condoms are suitable for steady relationships,” respectively (Table 3).

### 3.4. Factors Associated with Attitude toward Condom Use

The association between the independent variables and the dependent variable (attitude toward condom use) was tested using binary logistic regression analysis. Independent variables found statistically significant at *p* < 0.25 in the bivariate analysis were included in the multivariable binary logistic regression model. Finally, being a male, rural resident, ever had sex, and knowledge of STIs were significantly associated with a positive attitude toward condom use (Table 4).

## 4. Discussion

Young adults with good knowledge about condoms are still engaged in unsafe sexual practice [15]. To have safe sex, effective condom utilization is needed [14]. Condom use for safe sex is mainly influenced by attitudes toward condom use [22]. Based on the above scenario, we aimed to assess condom use and attitude toward condom use among college students in southwest Ethiopia. The proportion of positive attitude toward condom use was 53.4% (48.8%-58%). This study was in line with 52% in the Democratic Republic of Congo [19]. It was lower than 68.8% in Iraq [5].

The proportion of condom use was 66.1% (61.7%-70.5%). This study was in line with 68.3% in Ethiopia [20]. It was higher than 53% and 50.3% in Tanzania [12, 18], 24% in the Democratic Republic of Congo [19], 38.6% in Nigeria [6], 16.6% in Kenya [21], 12% in Iraq [5], and 51% in Spain [22]. The variation observed compared to other studies could be due to the differences in sample size, the operational definition used, and methodology in general. In addition, socioeconomic, behavioral/lifestyle, cultural, religious, and educational profiles of the study population may create a significant variation.

Sex was statistically associated with an attitude toward condom use in this study. Being male was associated with a positive attitude toward condom use. Male respondents were associated with 1.8 times increased odds of having a positive attitude toward condom use than female respondents. This could be due to the greater utilization of condoms by males (77.3%). Repeated condom use may create adaptation and a positive attitude to condom use. This study was consistent with studies conducted in Kenya and Tanzania [15, 18]. Besides, the association is better supported by the high prevalence of herpes simplex virus observed among females resulting from less attitude and practice toward condom use [32]. But a study conducted in Croatia suggests that women expressed a more positive attitude toward condom use than men [23].

Respondents who were from rural areas were associated with 2.2 times increased odds of having a positive attitude toward condom use than those from urban areas. Being from rural areas was associated with a positive attitude toward condom use. This finding was consistent with a study in Ethiopia [24].

In this study, ever had sex was statistically associated with a positive attitude toward condom use. Respondents who ever had sex were 1.9 times more likely to have a positive attitude toward condom use than those who did not. This finding was consistent with a study [16, 25]. This could be due to previous exposure to condoms which helps them develop a positive attitude toward condom use.

Respondents who had good knowledge of STIs were 1.7 times more likely to have a positive attitude toward condom use than poor knowledge of STIs. Good knowledge about STIs was associated with a positive attitude toward condom use. This finding was consistent with studies conducted in Kenya, Croatia, and Thailand [15, 23, 26]. But another study revealed that knowledge of sexually transmitted diseases, including HIV/AIDS and condoms, was not associated with more positive attitudes toward condom use or intention to use condoms with either steady or casual partners [16].

## 5. Conclusion

The proportion of positive attitude toward condom use among college students in Ethiopia was low. The study also found that being male, rural resident, ever had sex, and knowledge of STIs were factors associated with a positive attitude toward condom use. Therefore, strengthening information, education, and communication (IEC) on condom self-efficacy; providing condoms on campuses; and imparting education about sexually transmitted infections for young adults are central for improving condom use and attitudes toward condom use. Besides, inculcating sexual and reproductive health in the educational curriculum plays paramount importance.

## Figures and Tables

**Table 1:** Sociodemographic characteristics of the respondents at MAPtC in southwest Ethiopia.

Variables	Categories	Frequency	Percent
Sex	Male	243	53.6
	Female	210	46.4
Age	<20 years	217	47.9
	≥20 years	236	52.1
Religion	Orthodox	248	54.7
	Protestant	144	31.8
	Muslim	61	13.5
Marital status	Single	399	88.1
	Married	46	10.2
	Divorced	8	1.7
Residence	Rural	192	42.4
	Urban	261	57.6
Academic level	First year	51	11.3
	Second year	98	21.6
	Third year	180	39.7
	Fourth year	124	27.4

**Table 2:** Behavioral profiles of the respondents at MAPtC in southwest Ethiopia.

Variables	Categories	Frequency	Percent
Cigarette smoking (*n* = 453)	Yes	47	10.4
	No	406	89.6
Drinking alcohol (*n* = 453)	Yes	107	23.6
	No	346	76.4
Watching pornography (453)	Yes	172	38
	No	281	62
Sexually active (ever had sex)	Yes	180	39.7
	No	273	61.3
Condom use (*n* = 180)	Yes	119	66.1
	No	61	33.9
Number of sexual partners (*n* = 180)	<2	98	54.4
	≥2	82	45.6

**Table 3:** Attitude score of condom use among respondents at MAPtC in southwest Ethiopia.

Questions	Strongly disagree	Disagree	Not sure	Agree	Strongly agree
Condoms are an effective method of preventing pregnancy	68 (15%)	37 (8.2%)	125 (27.6%)	108 (23.8%)	115 (25.4%)
It is embarrassing for me to ask my partner to use a condom	87 (19.2%)	57 (12.6%)	111 (24.5%)	98 (21.6%)	100 (22.1%)
Condoms are suitable for casual sex	66 (14.6%)	30 (6.6%)	120 (26.5%)	105 (23.2%)	132 (29.1%)
Condoms are suitable for steady relationships	73 (16.7%)	60 (13.2%)	138 (30.5%)	85 (18.8%)	97 (21.4%)
It would be too embarrassing for me to buy or obtain condoms	99 (21.9%)	52 (11.5%)	119 (26.3%)	70 (15.5%)	113 (24.9%)
Condoms reduce sexual pleasure	76 (16.8%)	31 (6.8%)	155 (34.2%)	104 (23%)	87 (19.2%)
I feel protected while using a condom	82 (18.1%)	33 (7.3%)	126 (27.8%)	110 (24.3%)	102 (22.5%)
Condoms are too expensive to buy	101 (22.3%)	60 (13.2%)	133 (29.4%)	83 (18.3%)	76 (16.8%)
Condoms affect the mood in a negative way	102 (22.5%)	44 (9.7%)	160 (35.3%)	91 (20.1%)	56 (12.4%)
It is hard to tell my partner to use a condom if he/she does not want to use it	103 (22.7%)	69 (15.2%)	121 (26.7%)	89 (19.6%)	71 (15.7%)

**Table 4:** Factors associated with positive attitudes toward condom use of the respondents at MAPtC in southwest Ethiopia.

Variables	Categories	Attitude toward condom use		COR (95% CI)	AOR (95% CI)
		Negative	Positive		
Age group	<20 years	110	107	1	1
	≥20 years	101	135	1.37 (0.95-1.99)	1.06 (0.70-1.59)
Sex	Male	92	151	2.15 (1.47-3.13)	1.77 (1.19-2.65)^∗∗^
	Female	119	91	1	1
Residence	Rural	69	123	2.13 (1.45-3.12)	2.20 (1.47-3.30)^∗∗^
	Urban	142	119	1	1
Marital status	Single	180	190	1	1
	Married	16	33	2.38 (1.22-4.65)	1.70 (0.82-3.52)
	Divorced	15	19	0.56 (0.13-2.38)	0.40 (0.09-1.73)
Ever had sex	Yes	63	117	2.20 (1.49-3.24)	1.87 (1.23-2.85)^∗∗^
	No	148	125	1	1
Knowledge of STIs	Good	147	129	2.01 (1.37-2.96)	1.66 (1.10-2.51)^∗^
	Poor	64	113	1	1

CI = confidence interval; COR = crude odds ratio; AOR = adjusted odds ratio. ^∗^Significant at a *p* value < 0.05. ^∗∗^Significant at a *p* value < 0.01.

## Data Availability

The dataset is handled by the corresponding author and can be provided upon request.

## Abbreviations

AOR: Adjusted odds ratio
CI: Confidence interval
COR: Crude odds ratio
MAPtC: Mizan-Aman Polytechnic College
SPSS: Statistical Package for the Social Sciences
SD: Standard deviation
STIs: Sexually transmitted infections.

## Additional Points

*Limitations.* The nature of the study (cross-sectional study) may not show a cause-and-effect relationship. Besides, the possibility of social desirability bias may be considered a limitation to this study, since some instructors of the college served as a facilitator as well as a supervisor for the data collection process; the response of the respondents may resulted in either overestimate (respond more on good behavior) and underestimate (respond less on bad/undesirable behavior) report. This may shadow their response for interview questions.
